# Exosomal circRNAs contribute to intestinal development via the VEGF signalling pathway in human term and preterm colostrum

**DOI:** 10.18632/aging.202806

**Published:** 2021-04-04

**Authors:** Yahui Zhou, Zhangbin Yu, Xingyun Wang, Wenjuan Chen, Yiwen Liu, Yinghui Zhang, Jing Yin, Shuping Han

**Affiliations:** 1 Department of Pediatrics, Women’s Hospital of Nanjing Medical University, Nanjing Maternity and Child Health Care Hospital, Nanjing 210004, China; 2 Department of Pediatrics, Fourth Clinical Medicine College, Nanjing Medical University, Nanjing 210029, China

**Keywords:** human colostrum (HC), exosome, circRNAs, VEGF signaling pathway, intestine

## Abstract

Human breast milk (HBM) provides essential nutrients for newborn growth and development, and contains a variety of biologically active ingredients that can affect gastrointestinal tract and immune system development in breastfed infants. HBM also contains mRNAs, microRNAs and lncRNAs, most of which are encapsulated in milk-derived exosomes and exhibit various important infant development related biological functions. While previous studies have shown that exosomal circRNAs are involved in the intestinal epithelial cells’ proliferation and repair. However, the effect of HBM exosomal circRNAs on intestinal development is not clear. In this study, we identified 6756 circRNAs both in preterm colostrum (PC) and term colostrum (TC), of which 66 were upregulated, and 42 were downregulated *(|fold change>2|, p < 0.05)* in PC. Pathway analysis showed that the VEGF signalling pathway was involved, and network analysis revealed that the differentially expressed circRNAs bound various miRNAs. Further analyses showed that has_circRNA_405708 and has_circRNA_104707 were involved in the VEGF signalling pathway, and that they all bound various mirRNAs. Exosomes found in preterm colostrum (PC) and term colostrum (TC) promoted VEGF protein expression and induced the proliferation and migration of small intestinal epithelial cells (FHCs). Exosomal circRNAs found in human colostrum (HC) binding to related miRNAs may regulate VEGF signalling, and intestinal development.

## INTRODUCTION

Human breast milk (HBM), contains nutrients needed by infants as well as various non-nutritive bioactive ingredients that can promote growth and development [1]. Preterm milk (PM) is also known to contain more proteins and lipids than term milk (TM) [2, 3], and while there are many studies on the differences in macronutrients between PM and TM, there have only a few reports comparing exosomal content.

The immaturity of the intestines of premature babies means that they are particularly vulnerable to damage. Multiple studies have shown that breastfeeding significantly reduced necrotizing enterocolitis (NEC) [4, 5], and that breast milk (BM) can increase intestinal permeability, downregulate oxidative stress [6], and directly decrease damage intestinal epithelial cells [7].

Milk exosomes have important biological functions [8]. A previous study has shown that HBM exosomes reduce intestinal epithelial cell death [9] and promote intestinal epithelial cells growth [10]. There is emerging evidence that exosomal RNAs are involved in many processes, including cellular growth, antiviral activity, and insulin sensitivity [11].

CircRNAs were initially considered to be the product of abnormal RNA splicing [12], and many circRNAs have been discovered in recent years [13]. circRNAs have been shown to promote intestinal stem cell self-renewal [14], which is related to inflammatory bowel disease and intestinal barrier formation [15]. Recent studies have shown that exosomal circRNAs play critical roles in the development of neonatal tissue and organ, such as the brain [16] and the nervous system [17]. However, the expression and function of HBM exosomal circRNAs in intestinal development still remains unclear.

The aim of this study was to evaluate the differential expression of exosomal circRNAs in PC and TC and analyse key circRNAs that may regulate intestinal development. Differential expression of circRNAs between PC and TC groups was detected by microarray analysis, and 6756 exosomal circRNAs were identified. GO, pathway analyses and analysis of downstream miRNAs were used to identify and predict circRNAs functions. Our study may help to elucidate the role of HC in intestinal development.

## MATERIALS AND METHODS

### Ethics statement

HC was obtained from the Woman’s Hospital of Nanjing Medical University. This study was approved by the Institutional Review Board at the Women’s Hospital of Nanjing Medical University, Nanjing Maternity, and Child Health Care Hospital [Permission Number (2013)78].

### Human colostrum (HC) collection

Colostrum was collected from lactating mothers (n=18) who had donated their milk at the Woman’s Hospital of Nanjing Medical University. Nine had a full-term birth (≥37 weeks), and nine had preterm births (24-36 weeks). Colostrum was selected as our experimental sample material to reduce any differences that may be seen in human breast milk expressed at different times post birth, and to account for any differences between individual donors, each sample contained colostrum from there different donors. HBM was then obtained from these samples. The hospital provided the mother’s food, minimising the chance of food affecting colostrum composing between samples. Detailed data are shown in Table 1.

**Table 1:** Data related to the samples.

**Group**	**Sample**	**Mother**	**Age**	**Week**	**Weight**	**Delivery way**	**Parity**
		1	30	40+1	3430	natural birth	G2P2
	**T-1**	2	27	39+1	3180	natural birth	G1P1
		3	26	38+2	3310	natural birth	G1P1
		4	30	39	3600	Caesarean section	G2P2
**Term**	**T-2**	5	37	40+1	3640	natural birth	G2P1
		6	30	40+3	3590	natural birth	G2P2
		7	25	41+3	3740	natural birth	G2P1
	**T-3**	8	28	41+1	3550	natural birth	G1P1
		9	26	39+3	2800	natural birth	G1P1
	**P-1**	10	24	33	1870	Caesarean section	G1P1
		11	25	35+2	2730	natural birth	G1P1
		12	29	32	2120	natural birth	G1P1
		13	31	34+3	2480	natural birth	G2P1
**Preterm**	**P-2**	14	26	35+4	2460	natural birth	G1P1
		15	30	33+4	1870	natural birth	G2P1
		16	25	33+4	2290	natural birth	G3P1
	**P-3**	17	34	32+5	1830	Caesarean section	G3P2
		18	27	33+6	2790	natural birth	G4P2

### Isolation and purification of human colostrum exosomes

Exosomes were isolated immediately after colostrum samples were obtained from both breasts of each donor. The isolation method used in this study has been previously described [18]. In brief, 50 ml of HC was centrifuged twice at 3000 g for 15 min at 4° C to remove cells and fat globules. The supernatant was then transferred to new tubes, filtered through a 0.22 μm filter to remove any remaining debris and centrifuged at 42000 rpm at 4° C for 120 min. Exosomal pellets were stored at -80° C until use. The exosomes were observed with transmission electron microscopy (TEM) (USA, FEI Company, FEI Tecnai T20), and their quantity and size were assessed using the Nano Sight NS (Malvern, UK, 300 system, Nano Sight Technology). The collection time for all samples was within one month.

### Exosome labelling for *in vitro* studies

HC-Exo were labelled with PKH26 according to the manufacturer's protocol. The standard solution was passed through a 0.22μm filter (American microporous, Billerica's) and washed three times to remove excess dye. FHC cells were seeded in a 6-well plate for 6 h, then treated with PKH26 marker solution.

### Extraction and reverse transcription of total RNA

Total RNA was extracted from the samples using TRIzol Reagent (Invitrogen, Carlsbad CA, USA), and a pure tissue kit (Tiangen, DP431) was used to isolate the RNA. A 10 μM reverse transcription reaction was performed using 1000 ng of total RNA. The random primer reverse transcription method was used with the ScriptTM RT Master Mix kit, according to manufactures’ instructions, to generate cDNA synthesis for each sample.

### CircRNA extraction and microarray analysis

Arraystar Human circRNA Array v2 analysis was performed on the samples (Kangchen, Shanghai, China), and a NanoDrop ND-1000 was used to quantify otal RNA in each sample. The standard l Arraystar-based protocol was used for sample preparation and microarray hybridization. Briefly, RNase R (Epicentre, Inc.) was used to digest total RNA, remove linear RNAs, and enrich circular RNAs. We amplified and transcribed the enriched circRNAs into the fluorescent cRNA (Arraystar Super RNA Labelling Kit; Arraystar) and hybridized labelled cRNAs on the Arraystar Human circRNA Array v2 (8x15K, Arraystar). After washing, the array was scanned using an Agilent G2505C scanner.

The array image obtained by Agilent Feature Extraction software (version 11.0.1.1) was analyzed. Quantile normalization and subsequent data processing were performed using the LIMMA (implemented with the R software package). Volcanic maps were used to identify significant differences in the circRNA expression between the two groups and the hierarchical clustering method shows the distinguishable patterns of circRNA expression between samples. *|*log2 fold change (FC)| ≥ 2 and a *P* value < 0.05 by t test were considered to be statistically significant.

### qRT-PCR verification of target genes

qRT-PCR was used to evaluate the results of SYBR. The 2^-ΔΔCT^ method was used to analyse the experimental data. All data were averaged from three separate experiments. GAPDH was the internal reference for the target genes. Primer sequences are shown in Table 2.

**Table 2:** Primer sequences of circRNAs.

**Primer name**	**Sequence**
004239-F	ACCCAACAACCTGGTCCATA
004239-R	TGTTGGGGACCTTGTTCCTA
004239-F	ACCCAACAACCTGGTCCATA
004239-R	TGTTGGGGACCTTGTTCCTA
101018-F	GGCACAGTGAGACAGATGCT
101018-R	CATCATTTACAGTTTTTCCTGGTG
101018-F	GGCACAGTGAGACAGATGCT
101018-R	CATCATTTACAGTTTTTCCTGGTG
103356-F	GCATGATGCCTCATCAACAG
103356-R	GTCAAGTTCCTCCGACAAGC
GAPDH-F	GAAGGTGAAGGTCGGAGTC
GAPDH-R	GAAGATGGTGATGGGATTTC

### GO and KEGG pathway analysis

CircBase (http://www.circbase.org) was used to retrieve the encoded genes and predict their target genes. DAVID (https://david.ncifcrf.gov) was used for the target gene analysis.

### Western blotting analysis

Proteins were extracted for detection using the radioimmunoprecipitation (RIPA) test kit (Sigma-Aldrich, USA) according to the manufacturer's instructions and protein concentration measured using the bicinchoninic acid (BCA) toolkit (Pierce, USA). and SDS-containing polyacrylamide gels (SDS-PAGE) were used to separate equal amounts of protein Samples (30 μg for exosome pellets). These samples were then transferred to a polyvinylidene fluoride (PVDF) membrane (Bio-Rad, USA). Then, 1 x TBST (0.1 M, pH 7.4) containing 5% skim milk was used to block the membrane for 1 h, hen hybridized with CD9 (Proteintech, 1:1000 dilution), CD63 (Proteintech, 1:1000 dilution), CD81 (Proteintech, 1:1000 dilution), PI3K/P-PI3K (Proteintech, 1:1000 dilution), AKT/P-AKT (Proteintech, 1:1000 dilution) and VEGF (Proteintech, 1:1000 dilution) antibodies. The immune complex was incubated with a secondary antibody conjugated to horseradish peroxidase (Applygen, China; 1:5000 dilution) for 1 h at room temperature. Protein bands were examined and the signal intensities of interest were calculated using ImageJ software (National Institutes of Health, USA). The results are presented as the relative fold change ± SD.

### Cell proliferation assay

Cell proliferation was assessed using the Cell Counting Kit-8 (CCK-8) assay (Dojindo, Tokyo, Japan) according to the manufacturer's instructions. FHC cells (X-Y Biotechnology) were grown in 96-well plates with a cell density of approximately 1x10^3^ cells per well then co-cultured with TM or PM exosomes (20.0 μg of protein) for 0, 12, 24, or 36 h. The cells were measured at 450 nm (optical density (OD) value) using a microplate reader (BioTek Instruments, Inc., Germany).

### Wound closure assay

FHC cells were plated into a 6-well plates and scratched with the tip of a sterile pipette. TM and PM exosomes were added (200 μg). The scratched areas were imaged under a microscope at 0 and 12 h, and ZEN software (Zeiss, Germany) was used to measure the scratches widths.

### Statistical analysis

Data were analysed by SPSS, and Student’s t-test or one-way ANOVA was employed for statistical comparisons. *P < 0.05* was statistically significant.

## RESULTS

### Characteristics of the HC exosomes

**Figure 1:**
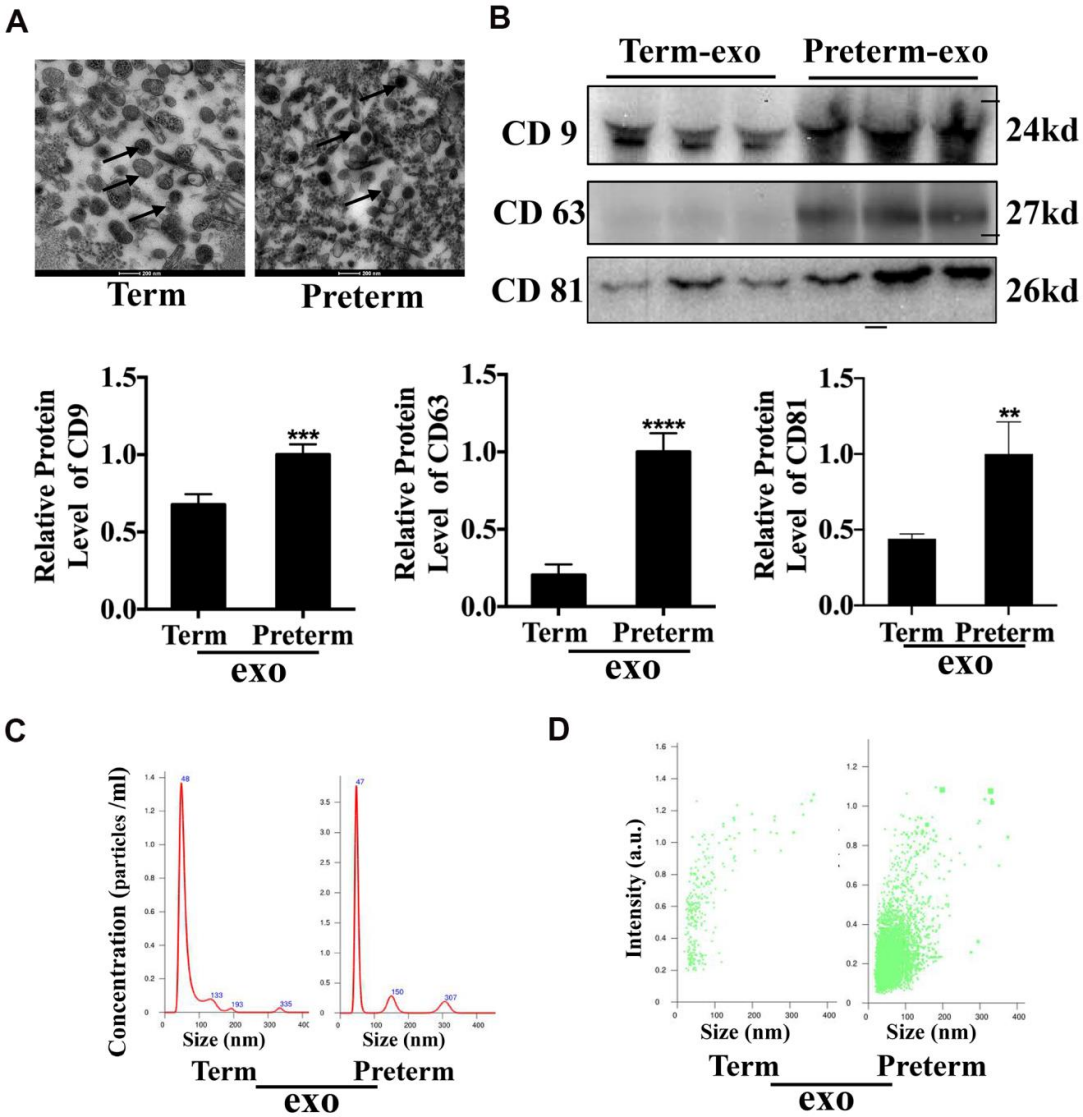
HC Exosome characterization (**A**) TEM observation of breast milk exosomes showed a typical cup-shaped structure. (**B**) WB verification of presence of CD9, CD81 and CD63. (**C**) NTA analysis of preterm and term milk exosomes diameters. (**D**) Comparison of preterm and term breast milk exosomes abundance.

Exosomes were isolated from human TC and PC using ultracentrifugation and transmission electron microscopy (TEM) was used to investigate them at the nanometre scale (Figure 1A). CD63, CD9 and CD81 are exosome surface markers and all were expressed in PM. CD81 and CD9 were also expressed in the TM exosomes, although CD81 expression was lower in TC exosomes compared to PC exosomes (Figure 1B). Nanosight (NTA) analysis showed that TC and PC exosomes were both 40-50 nm in diameter (Figure 1C) and there was a significantly higher abundance of exosomes in PM compared to TM (Figure 1D). These results suggest that exosomes were have been successfully isolated from the TC and the PC, and that the exosome levels may be higher in the PC than TC.

### Differentially expressed circRNAs in HC exosomes

**Figure 2:**
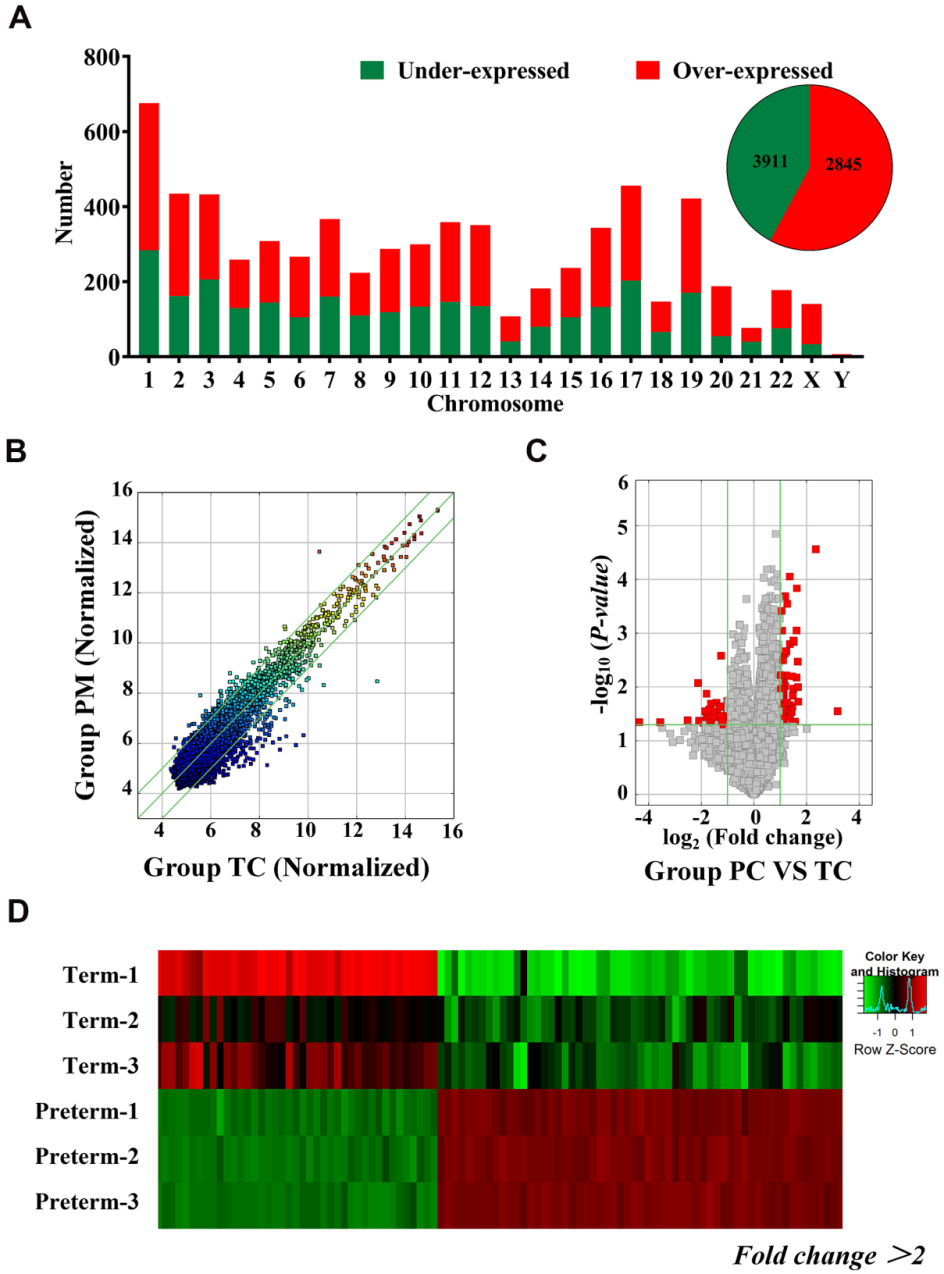
**Differentially expressed circRNAs in HC exosomes.** (**A**) A total of 6756 circRNAs were detected, 3911 were upregulated and 2845 were downregulated. Histogram showing the differentially expressed circRNAs in the human chromosome. (**B**) The X-axis and Y-axis values are normalized signal values (log2 scaling). The green line represents a broken line. Above and below the upper and lower green lines, the circRNAs exhibited more than 2.0-fold changes between the preterm milk (PM) and term milk (TM) groups. (**C**) The red dots in the volcano map represent statistically significant differentially expressed circRNAs. (**D**) Heat map of circRNA results from the PC and TC groups. Red bars indicate higher expression, and green bars indicate lower expression.

Exosomal circRNAs from TC and the PC were identified through microarray analysis. A total of 6756 circRNAs were detected and 2845 were downregulated and 3911 circRNAs were upregulated in PC. These differentially expressed circRNAs were distributed across all chromosomes, including sex chromosomes (Figure 2A). Detailed information on the differentially expressed circRNAs is shown in Supplementary Table 1. Detected circRNAs are also shown by scatter plots and volcano maps (Figure 2B, 2C), and a heat map illustrates the differences in circRNAs, 66 of which were upregulated and 42 were downregulated (fold change (FD) > 2, P < 0.05) (Figure 2D).

### Verification of circRNAs by qRT-PCR

**Figure 3:**
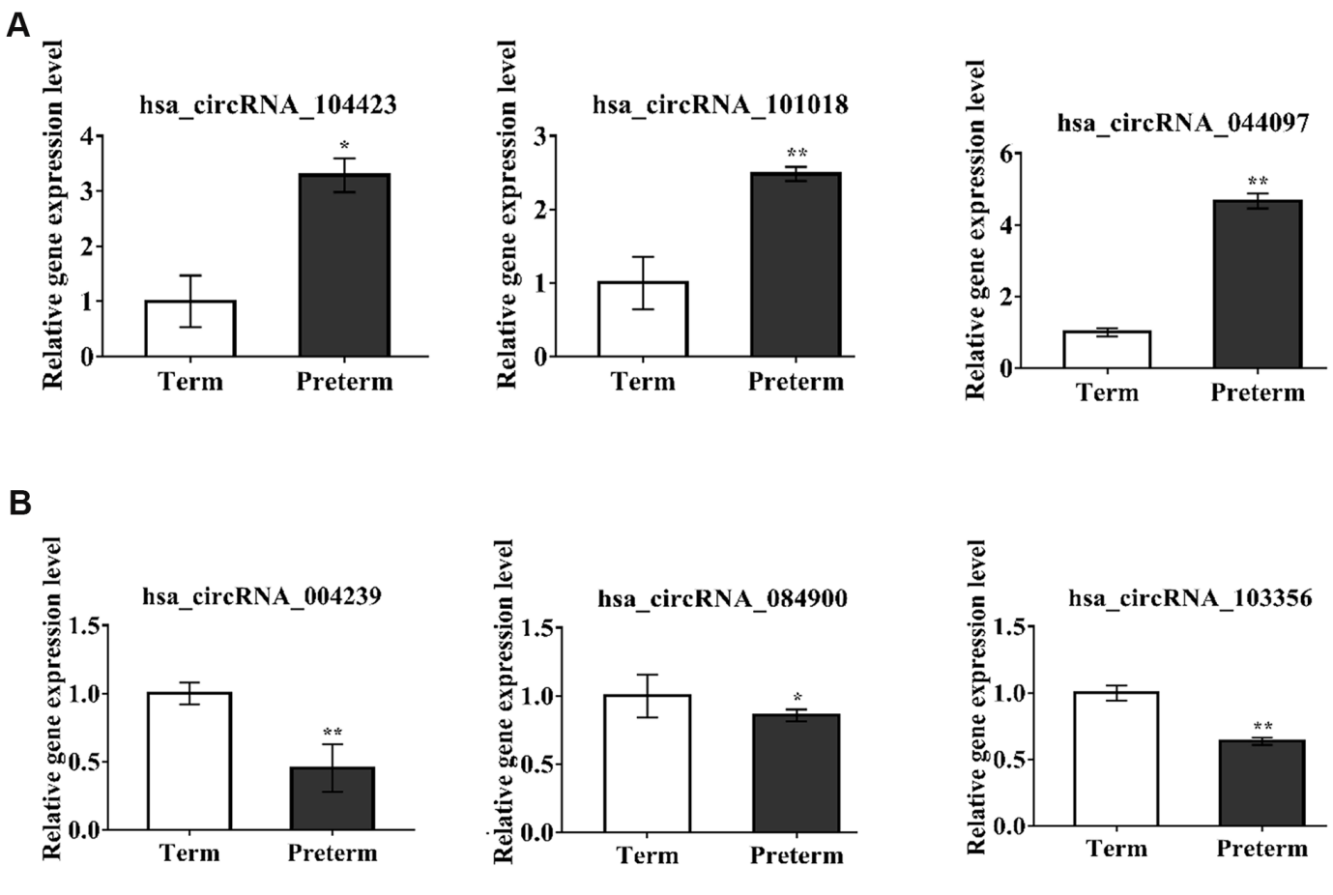
**circRNAs expression verification by qPCR.** *P-value < 0.05, **P < 0.01 ***P < 0.001. (**A**) The circRNAs upregulated in PC, (**B**) The circRNAs downregulated in PC.

To verify the exosomal circRNAs which were significantly expressed in the PC and TC, we randomly selected 6 differentially expressed circRNAs for qRT-PCR verification in the same samples used for the HC microarray analysis; 3 upregulated (hsa_circRNA_104423, hsa_circRNA_101018, hsa_circRNA_044097) and 3 downregulated (hsa_circRNA_004239, hsa_circRNA_084900, hsa_circRNA_103356) circRNAs were selected (Figure 3A, 3B). The results showed that verified circRNAs expression was consistent with the microarray analysis results.

### GO enrichment and KEGG signal pathway analysis

**Figure 4:**
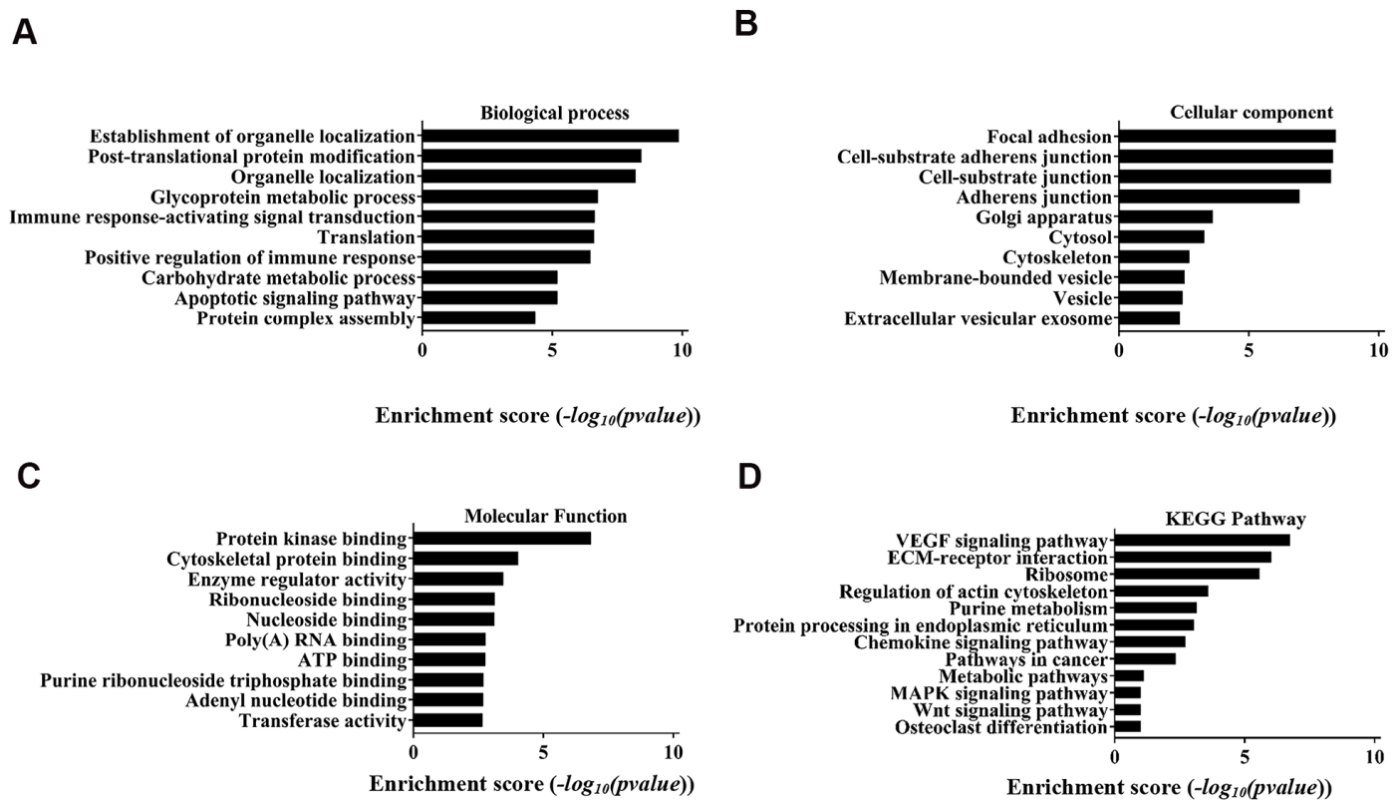
**Top 10 GO terms with gene count and enrichment analysis for biological processes.** (**A**) Biological process, (**B**) Cellular component, (**C**) Molecular function. (**D**) The top 10 enriched KEGG pathways of the parental genes of the differentially expressed circRNAs.

GO enrichment and signal pathway analysis were performed to assess potential functions. Biological process analysis showed that the circRNAs were mostly involved in organelle localization, post-translational protein modification, glycoprotein metabolic process, immune response-activating signal transduction translation, positive regulation of immune response, carbohydrate metabolic process, apoptotic signalling pathway, and protein complex assembly (Figure 4A). The cellular component analysis demonstrated that the potential functions were significantly related to focal adhesion, cell-substrate junction, Golgi apparatus, cytosol, cytoskeleton, membrane-bound vesicle, vesicle and extracellular vesicular exosomes (Figure 4B). Molecular function indicated that the most significant functions were protein kinase binding, cytoskeletal protein binding, enzyme regulator activity, ribonucleoside binding, nucleoside binding, poly(A) RNA binding, ATP binding purine ribonucleoside triphosphate binding, adenyl nucleotide binding and transferase activity (Figure 4C). Analysis of the KEGG pathway showed that the VEGF signalling pathway was the most significant pathway.

### Detailed annotation of the interaction between circRNAs and miRNAs

**Figure 5:**
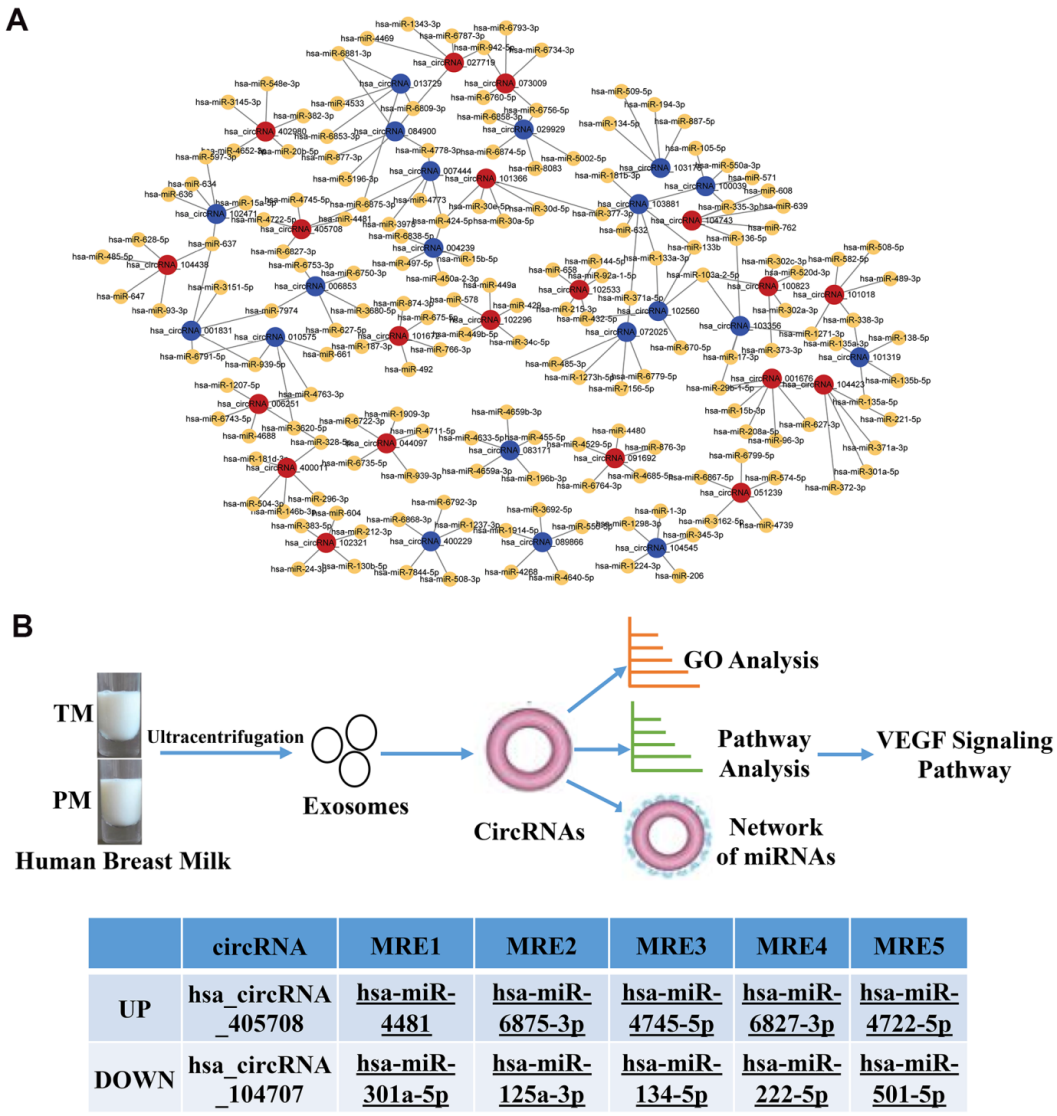
**Network of the relationship between circRNAs and miRNAs.** (**A**) Red dots represent upregulated circRNAs in PC, blue dots represent downregulated circRNAs in PC, and yellow dots represent downstream miRNAs that bind to circRNAs; (**B**) Flow chart of the experiment and two circRNAs involved in the VEGF signalling pathway, one of which was upregulated and the other was downregulated.

Previous studies found that circRNAs perform biological functions through their downstream miRNAs. To evaluate the target miRNAs, Target Scan and miRanda databases were used to make theoretical predictions based on conservative seed matching sequences. 108 circRNAs (FD>2.0, P<0.05) were identified that could bind to miRNAs (Supplementary Table 2). The relationship between the circRNAs and miRNAs is shown as a network (top 20 differentially expressed circRNAs) (Figure 5A). The KEGG signalling pathway analysis showed that the VEGF signalling pathway was the most significantly enriched. Further analysis identified two circRNAs involved in the VEGF signalling pathway, one of which was upregulated and the other was downregulated. They bound to the miRNAs hsa_miR-4481, hsa_miR-6875-3p, hsa_miR-4745-5p, hsa_miR-6827-38, hsa_miR-4722-5p, hsa_miR-301a-5p, hsa_miR-125a-3p, hsa_miR-134-5p, hsa_miR-222-5p and hsa_miR-501-5p (Figure 5B).

### HC exosomes function in small intestinal epithelial cells (FHCs) is through the VEGF signalling pathway

**Figure 6:**
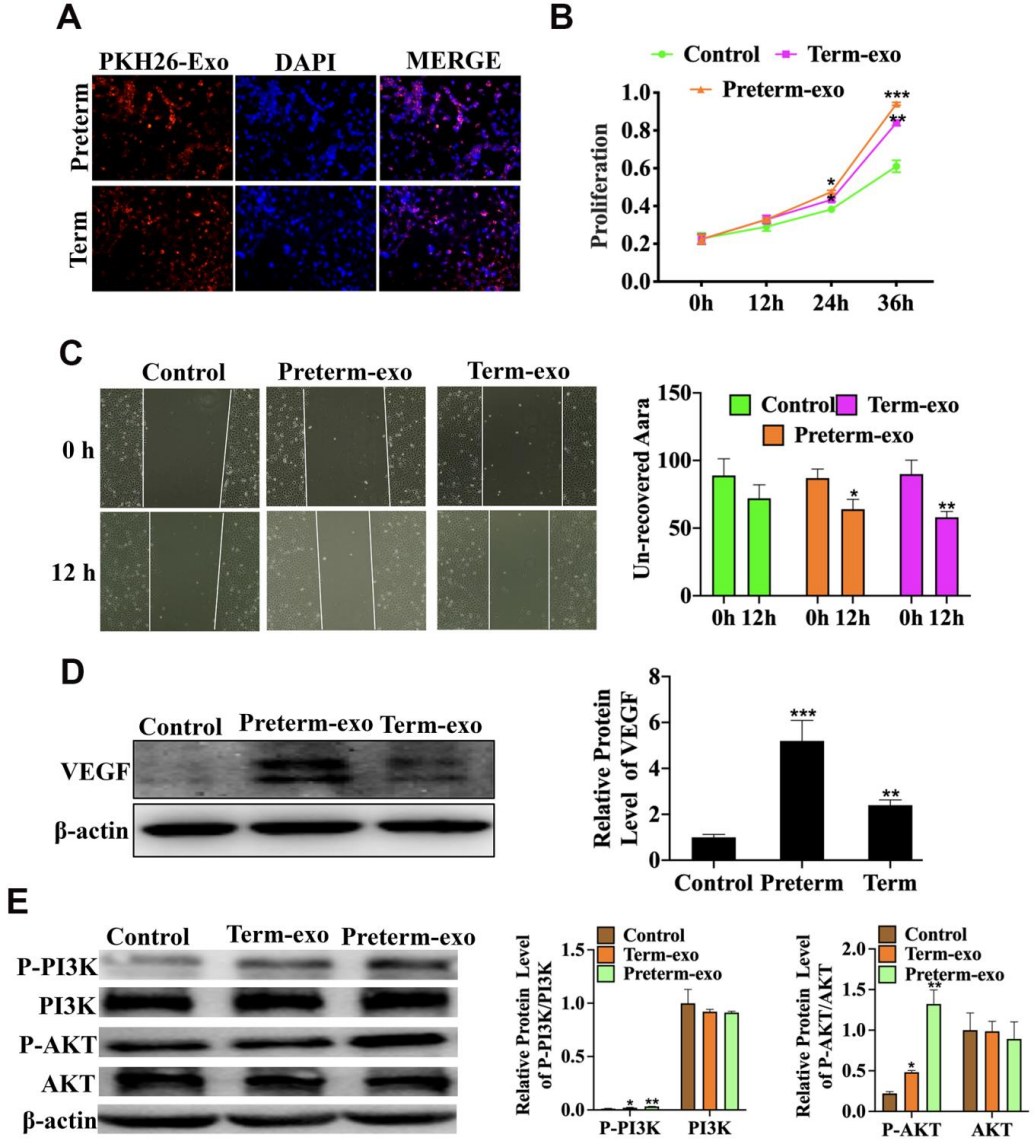
**HC exosomes function through the VEGF signalling pathway in small intestinal epithelial cells (FHCs).** (**A**) PKH26 labelled exosomes taken up into the cells, (**B**) Proliferation of intestinal epithelial cells (FHC); (**C**) the migration of intestinal epithelial cells (FHC); (**D**) the VEGF protein expression; (**E**) P-PI3K/PI3K and P-AKT/AKT protein expression.

The KEGG signalling pathway analysis showed that the VEGF signalling pathway was the most significantly enriched. Further functional experiments have shown that HC-Exo, taken up by FHC cells after PKH26 labelling for 6h, could promote proliferation and migration of intestinal epithelial cells (FHC) (Figure 6A–6C) and that the PM exosomes were significantly more potent than the TM exosomes. Experiments have also shown that PC and TC exosomes promoted the VEGF protein expression (Figure 6C) and phosphorylation of PI3K and AKT in FHC cells, which are the key downstream regulators of VEGF signalling. PI3K phosphorylation was higher with PC than TC exosomes (Figure 6D).

### VEGFR inhibition blocked the proliferation and migration of FHC cells induced by PC and TC exosomes

**Figure 7:**
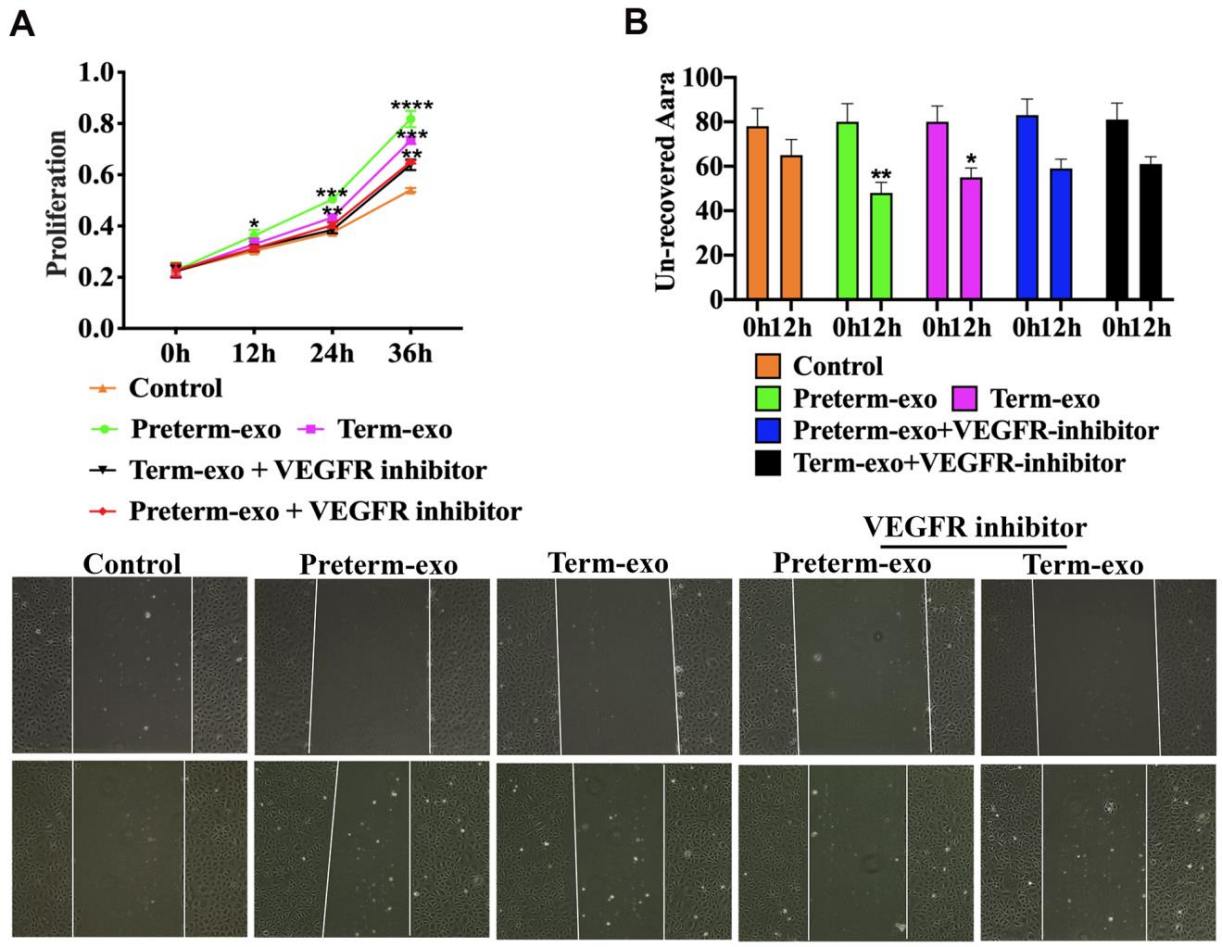
**VEGFR inhibition blocks PC and TC exosome-induced FHC proliferation and migration.** (**A**) TM and PM exosomes-induced proliferation, and (**B**) migration of FHC cells after VEGFR inhibitor intervention.

AV951 (a VEGFR inhibitor) was added to suppress the VEGR signalling to further verify the HC-Exo involvement in the pathway. It was shown that inhibition of VEGF signalling significantly decreased the proliferation and migration of FHC cells promoted by TC and PC exosomes (Figure 7A, 7B).

## DISCUSSION

Because it contains many active substances, which are particularly important for the growth and development of newborns, HBM quality is especially important for premature infants [1, 19]. PM and TM compositions are significantly different, but they had significant biological function [3]. To an extent, milk's nutritional content is related to the mother's diet [20]. HBM used in this study was from the colostrum of the mothers of newborns During the collection period, our hospital food department provided the mothers' food, which reduced the HBM content variation to some extent. Previous studies have shown that breast milk reduces the risk of NEC [7]. The abundant bioactive factors in HBM are essential for the development of the innate immune system of the gastrointestinal tract [21], and a number of studies have shown that BM exosomes may affect intestinal development. For example, intestinal epithelial cells growth in infants can be promoted by BM exosomes. [10, 22]. Inhibition of the TLR4/NF-κB and p53 pathways attenuated LPS-induced intestinal epithelial cell apoptosis [23] and promoted cell proliferation [24]. In this study, TEM, Nano-sight, and surface markers (CD63, CD9 and CD81) analysis has shown that we successfully extracted HC-Exo. It was observed that CD63 and CD81 levels were lower in TC exosomes than in PC, which may be due to there being fewer exosomes in TC than in PC.

Exosomes contain many active ingredients (including miRNAs, lncRNAs and mRNAs) [25, 26], as well as new types of long noncoding RNAs (circRNAs) [13]. Recent studies have suggested that exosomal miRNAs extracted from milk promote commitment of Treg cells commitment by decreasing the immune response of Il-4/Th2-mediated allergic sensitization and that milk transfer of exogenous miR-155 may induce thymic regulatory T cells to secrete key components required for immune regulation and epigenetic modification [27]. Intestinal immune system maturation is crucial for intestine developing, and these RNAs may strengthen the immune system and, in turn, promote development. The roles of exosomal circRNAs involved in intestinal development remain unclear. In this study, we isolated circRNAs from PC and TC exosomes and analysed them by high-throughput microarray screening.

We performed GO analysis and found that differentially expressed circRNAs in PC and the TC exosomes significantly participated in immune response, activation of signal transduction, translation, and positive regulation of the immune response. The immune response and immune activation play roles in newborns development [28], and by improving the intestinal immune system of newborns, help to promote resistance to bacterial infection [29, 30]. The *in vitro* synthesis of circRNAs activates the innate immune response [31], which protects against viral infection. In addition, the immune factors NF90/NF110 regulate circRNA biosynthesis and inhibit viral infection by interacting with viral miRNAs [31, 32]. These findings indicate that exosomal circRNAs from HC may be critical in the development of the immune system of newborns and thus promote intestinal development and help defend against neonatal infection.

The VEGF signalling pathway regulates angiogenesis of various organs and tissues and alleviates adaptive cerebral ischaemia/reperfusion injury in rats [33]. Exosomal MMP2 from mature osteoblasts promotes endothelial cell angiogenesis through the VEGF signalling pathway [34]. The intestinal microvascular system is critical in NEC [35]. In neonatal mice, loss of VEGFR2 signalling led to dysplasia of the intestinal microvascular system and necrotizing enterocolitis in neonatal mice. Inhibition of VEGFR2 kinase activity also reduced villous endothelial cells proliferation in newborns mice and increased morbidity and mortality from NEC [36]. In this study, we found that the primary enrichment pathway of exosomal circRNAs of the PC and the TC was the VEGF signalling pathway. We also showed in FHCs, PC and TC exosomes can promote cell proliferation and migration, and that VEGF protein expression was also increased. PC and TC exosomal circRNAs may therefore promote intestinal epithelial cell proliferation and migration through the VEGF signalling pathway.

Recent studies have also shown that circRNAs can bind miRNA and regulate downstream miRNAs, all of which are involved in various pathological mechanisms. For example, studies conducted by Eur Heart J et al. showed that the cardiac circRNA HRCR could bind directly binds to miR-223 and inhibits HRCR expression in human cardiomyocytes, producing a hypertrophic response [37], and ciRS-7 (human circRNA cerebellar degeneration-related protein 1 transcript (CDR1)) binding to related miRNAs can affect the availability of miR-7 to bind to its target mRNAs [38–40]. CircSRY also acts as a sponge of miR-138 and as a platform for the binding of miR-138, regulating the mRNA translation of its targets [41]. Sponging of 9 miRNAs, including miR-124, which regulates cell growth, has been previously reported [42]. In our study, we predicted that exosomal circRNAs derived from the PC and the TC bind various miRNAs, some of which have been shown to regulate the physiology and pathology of intestinal development. For example, hsa_circ_0000726 binds miRNA181 and reduces HK and TNF-α expression, inducing glycometabolism and the inflammatory response, respectively [43]. Hsa_circ_083171 binds to miRNA-455, and miR-455-5p downregulates STRA6, reducing cell proliferation [44] and alleviating apoptosis, oxidative stress and the inflammatory response [45]. MiR-455-5p can also alleviate oxygen-induced myocardial cell injury [46]. This study indicated that the exosomal circRNAs derived from HBM might regulate these miRNAs and promote intestinal development. After further analysis, two exosomal circRNAs derived from PC and TC, hsa_circRNA_405708 and hsa_circRNA_104707, respectively, were found to be involved in the VEGF signalling pathway. It was previous reported that circRNAs, such as circ_001621, can sponges the miR-578 and regulate VEGF expression to promote osteosarcoma cell proliferation and migration [47]. These two exosomal circRNAs also bind to many miRNAs, including hsa-miR-4481, hsa-miR-6875-3p, hsa-miR-4745-5p hsa-miR-6827-3p, hsa-miR-4722-5p, hsa-miR-301a-5p, hsa-miR-125a-3p, hsa-miR-134-5p hsa-miR-222-5p and hsa-miR-501-5p. Our results indicated that the PM and TM derived exosomal circRNAs might bind miRNAs, regulate the VEGF signalling pathway, and then influence intestinal development.

Overall, circRNAs have an important effect on multiple pathways in the intestine and should be the focus of future studies. Further research is also needed to validate the potential function of these exosomal circRNAs and their mechanism in intestinal development.

## CONCLUSIONS

This study confirmed that there were significant differences in PC and TC derived exosomal circRNAs, and that these circRNAs may play important biological roles through their downstream miRNAs. KEGG pathway analysis identified the significant enrichment of the VEGF signalling pathway, and it was shown that PC and TC exosomes can promote the proliferation and migration of FHC cells and VEGF expression. HC exosomes may promote cell proliferation and migration through the VEGF signalling pathway, thereby promoting intestinal development.

### Ethics approval and consent to participate

The study was approved by the Institutional Review Board at Women’s Hospital of Nanjing Medical University, Nanjing Maternity and Child Health Care Hospital [Permission Number (2013)78]. Written consent was obtained from all participants.

### Data availability statement

The date that support the findings of this study are available from the corresponding author upon reasonable request.

## Supplementary Material

Supplementary Table 1

Supplementary Table 2
