# Supplementary material for: Exosomal circRNAs contribute to intestinal development via the VEGF signalling pathway in human term and preterm colostrum
Source: Aging (Albany NY). 2021 Apr 4;13(8):11218–33. doi: 10.18632/aging.202806 (PMC8109075; doi:10.18632/aging.202806)
Supplement: Supplementary Table 1 [file aging-13-202806-s001.doc]

**Supplementary Table 1.** **The most differential expressed circRNAs.**

| **circRNA up** | **GeneSymbol** | **FC(abs)** | **P-value** |
| --- | --- | --- | --- |
| hsa_circRNA_405708 | NFATC1 | 9.0734664 | 0.028059514 |
| hsa_circRNA_400011 | GADD45A | 5.0877751 | 2.73252E-05 |
| hsa_circRNA_104299 | USP42 | 4.0235423 | 0.059657669 |
| hsa_circRNA_073009 | ENC1 | 3.2025458 | 0.018636864 |
| hsa_circRNA_100823 | LPXN | 3.1915371 | 0.010000778 |
| hsa_circRNA_102321 | CABLES1 | 3.1836116 | 0.003370229 |
| hsa_circRNA_104438 | AP4M1 | 3.0962513 | 0.000146573 |
| hsa_circRNA_027719 | SOCS2-AS1 | 3.0711469 | 0.00665509 |
| hsa_circRNA_402980 | ATP1B3 | 3.0677124 | 0.000889646 |
| hsa_circRNA_104743 | MLLT3 | 2.9559398 | 0.043684434 |
| hsa_circRNA_404578 | PRPF3 | 2.9168323 | 0.068943478 |
| hsa_circRNA_101672 | RAB40C | 2.8499296 | 0.001446797 |
| hsa_circRNA_001676 | PPP1R12A | 2.8309804 | 0.001368584 |
| hsa_circRNA_091692 | CD99L2 | 2.8162426 | 0.010782514 |
| hsa_circRNA_010027 | PDPN | 2.7975591 | 0.053837694 |
| hsa_circRNA_102296 | ANKRD12 | 2.7820591 | 0.014540657 |
| hsa_circRNA_051239 | ATP5SL | 2.7627001 | 0.022996403 |
| hsa_circRNA_102533 | UBA2 | 2.7290675 | 0.045014518 |
| hsa_circRNA_101366 | ZBTB25 | 2.7274333 | 0.006302046 |
| hsa_circRNA_006251 | TET3 | 2.6854109 | 0.028851265 |
| hsa_circRNA_100836 | BSCL2 | 2.6807141 | 0.053097691 |
| hsa_circRNA_100387 | PRRC2C | 2.6195955 | 0.053103253 |
| hsa_circRNA_404905 | INPPL1 | 2.5946365 | 0.031975921 |
| hsa_circRNA_029965 | PDS5B | 2.580745 | 0.021525508 |
| hsa_circRNA_061110 | RPS21 | 2.5747022 | 0.001585979 |
| hsa_circRNA_081426 | AP4M1 | 2.5621408 | 8.81515E-05 |
| hsa_circRNA_101018 | ATF7IP | 2.4882464 | 0.023499948 |
| hsa_circRNA_104338 | CREB5 | 2.4615858 | 0.051278285 |
| hsa_circRNA_404736 | KIAA1462 | 2.4262103 | 0.035026295 |
| hsa_circRNA_003550 | WDR1 | 2.4230672 | 0.054830522 |
| hsa_circRNA_100780 | QSER1 | 2.4055262 | 0.030010588 |
| hsa_circRNA_406167 | COL6A2 | 2.3967052 | 0.000283134 |
| hsa_circRNA_400005 | RPS6KA1 | 2.3865072 | 0.019338271 |
| hsa_circRNA_102731 | XPO1 | 2.3688085 | 0.054864273 |
| hsa_circRNA_400082 | PLEKHG4B | 2.3579007 | 0.071649659 |
| hsa_circRNA_405450 | C16orf62 | 2.3446124 | 0.002174311 |
| hsa_circRNA_400229 | OSBPL9 | 2.3433843 | 0.006040221 |
| hsa_circRNA_406295 | SUCLG2-AS1 | 2.3188133 | 0.02525355 |
| hsa_circRNA_087631 | HABP4 | 2.2871352 | 0.069633016 |
| hsa_circRNA_402893 | PTPRG | 2.2784832 | 0.009632376 |
| hsa_circRNA_100691 | HSPA12A | 2.277131 | 0.000206375 |
| hsa_circRNA_087298 | TLE4 | 2.266086 | 0.116975967 |
| hsa_circRNA_103179 | CABIN1 | 2.2498493 | 0.00238515 |
| hsa_circRNA_102788 | NPAS2 | 2.2417435 | 0.002150433 |
| hsa_circRNA_406165 | COL6A2 | 2.236216 | 0.022056663 |
| hsa_circRNA_059571 | RALGAPA2 | 2.2298508 | 0.262354997 |
| hsa_circRNA_001038 | BCL7A | 2.2246878 | 0.054301863 |
| hsa_circRNA_104238 | TIAM2 | 2.2220177 | 0.032615022 |
| hsa_circRNA_004566 | ANKRD11 | 2.2143026 | 0.022749224 |
| hsa_circRNA_406313 | TFG | 2.2137033 | 0.045839884 |
| hsa_circRNA_100832 | FADS1 | 2.2107521 | 0.007010916 |
| hsa_circRNA_084900 | KIAA1429 | 2.2067187 | 0.035887351 |
| hsa_circRNA_029929 | BRCA2 | 2.1930395 | 0.018538341 |
| hsa_circRNA_014205 | S100A11 | 2.1853763 | 0.013430381 |
| hsa_circRNA_036188 | PKM | 2.1770538 | 0.037545316 |
| hsa_circRNA_029930 | BRCA2 | 2.1747558 | 0.01137735 |
| hsa_circRNA_050263 | ATP13A1 | 2.1703851 | 0.042228338 |
| hsa_circRNA_102101 | CDC27 | 2.1648989 | 0.180465721 |
| hsa_circRNA_401876 | DNAI2 | 2.1603313 | 0.015212528 |
| hsa_circRNA_005310 | SBNO1 | 2.1602767 | 0.003184008 |
| hsa_circRNA_000620 | ANKRD11 | 2.1556935 | 0.027429672 |
| hsa_circRNA_100114 | RPA2 | 2.138856 | 0.026377517 |
| hsa_circRNA_101089 | MARS | 2.132197 | 0.011627592 |
| hsa_circRNA_402197 | SLC30A6 | 2.1305967 | 0.011238255 |
| hsa_circRNA_104798 | TLE4 | 2.1247789 | 0.036255889 |
| hsa_circRNA_101796 | ASPHD1 | 2.1213337 | 0.059964085 |
| hsa_circRNA_004036 |  | 2.1098273 | 0.130288926 |
| hsa_circRNA_402942 | ITGB5 | 2.1086668 | 0.032262425 |
| hsa_circRNA_402500 | IQCA1 | 2.1064202 | 0.061206957 |
| hsa_circRNA_061170 | RTEL1 | 2.0951679 | 0.191949704 |
| hsa_circRNA_052372 | TRIM28 | 2.091545 | 0.014584303 |
| hsa_circRNA_014225 | S100A6 | 2.0893682 | 0.000899748 |
| hsa_circRNA_004239 | C17orf53 | 2.0807202 | 0.027201666 |
| hsa_circRNA_007771 | LRP11 | 2.070065 | 0.000381879 |
| hsa_circRNA_406457 | SEPSECS-AS1 | 2.0595961 | 0.083817809 |
| hsa_circRNA_102520 | UBA2 | 2.0593659 | 0.111064714 |
| hsa_circRNA_000361 | PLCL2 | 2.0576243 | 0.067296283 |
| hsa_circRNA_060102 | ERGIC3 | 2.0574108 | 0.159712949 |
| hsa_circRNA_013141 | TGFBR3 | 2.0528567 | 0.014365375 |
| hsa_circRNA_076700 | TNFRSF21 | 2.0507097 | 0.006798411 |
| hsa_circRNA_104423 | CDK14 | 2.0487171 | 0.006678272 |
| hsa_circRNA_103355 | SMARCC1 | 2.047051 | 0.02098957 |
| hsa_circRNA_100988 | FLI1 | 2.0451621 | 0.010264795 |
| hsa_circRNA_104412 | HIP1 | 2.0405826 | 0.010964867 |
| hsa_circRNA_035301 | MAPK6 | 2.0353074 | 0.023828578 |
| hsa_circRNA_001788 | PROSC | 2.0332262 | 0.056256864 |
| hsa_circRNA_103735 | MFSD8 | 2.0279508 | 0.006018434 |
| hsa_circRNA_104797 | TLE4 | 2.0276181 | 0.066654177 |
| hsa_circRNA_401403 | PATL2 | 2.0261431 | 0.063036194 |
| hsa_circRNA_104475 | FAM71F2 | 2.0260546 | 0.007827217 |
| hsa_circRNA_060184 | MYL9 | 2.0200262 | 0.106292616 |
| hsa_circRNA_055426 | GGCX | 2.015722 | 0.119929428 |
| hsa_circRNA_103356 | SMARCC1 | 2.0140925 | 0.023535815 |
| hsa_circRNA_103145 | ADARB1 | 2.0069087 | 0.03299435 |
| **circRNA down** | **GeneSymbol** | **FC (abs)** | **P-value** |
| hsa_circRNA_001831 | SCRIB | 20.6594208 | 0.044982909 |
| hsa_circRNA_010575 | HSPG2 | 11.8597859 | 0.045220758 |
| hsa_circRNA_405463 | ZNF423 | 11.2044525 | 0.057240536 |
| hsa_circRNA_101833 | DUS2 | 9.0819763 | 0.072656064 |
| hsa_circRNA_404013 | BRF2 | 8.5306862 | 0.105503329 |
| hsa_circRNA_103670 | CNOT6L | 7.0623081 | 0.096201641 |
| hsa_circRNA_103809 | ZFR | 6.2503239 | 0.066019518 |
| hsa_circRNA_102471 | MYO9B | 5.7194571 | 0.041322837 |
| hsa_circRNA_085362 | TRPS1 | 5.5104532 | 0.056243871 |
| hsa_circRNA_104168 | RTN4IP1 | 5.2532268 | 0.106140457 |
| hsa_circRNA_100191 | PTPRF | 4.9750161 | 0.185238774 |
| hsa_circRNA_104052 | CDYL | 4.8283843 | 0.058455317 |
| hsa_circRNA_403876 | PDAP1 | 4.7280016 | 0.085228622 |
| hsa_circRNA_031896 | TMX1 | 4.7098916 | 0.132348952 |
| hsa_circRNA_001937 | CHD9 | 4.6636555 | 0.091666169 |
| hsa_circRNA_007444 | RHOBTB3 | 4.3578619 | 0.00837232 |
| hsa_circRNA_006853 | RPPH1 | 4.267976 | 0.043037677 |
| hsa_circRNA_104852 | RAD23B | 4.2674452 | 0.107736262 |
| hsa_circRNA_100013 | GNB1 | 4.2121805 | 0.097810625 |
| hsa_circRNA_089866 | WWC3 | 4.2004572 | 0.042253083 |
| hsa_circRNA_036592 | SCAND2P | 4.1815732 | 0.080822114 |
| hsa_circRNA_100313 | MAN1A2 | 3.9174548 | 0.093972394 |
| hsa_circRNA_013729 | MAN1A2 | 3.8051867 | 0.046157007 |
| hsa_circRNA_100146 | EIF3I | 3.7651439 | 0.129419737 |
| hsa_circRNA_026074 | ARF3 | 3.7590107 | 0.130488672 |
| hsa_circRNA_100018 | GNB1 | 3.7097075 | 0.188597048 |
| hsa_circRNA_044097 | EFTUD2 | 3.6149661 | 0.028188721 |
| hsa_circRNA_059914 | AHCY | 3.5953247 | 0.178620297 |
| hsa_circRNA_000120 | MAN1A2 | 3.5751716 | 0.078753902 |
| hsa_circRNA_104566 | PSD3 | 3.5715777 | 0.050104714 |
| hsa_circRNA_104708 | PTK2 | 3.5671699 | 0.057691617 |
| hsa_circRNA_104980 | WWC3 | 3.5450597 | 0.077477555 |
| hsa_circRNA_100039 | RERE | 3.4838673 | 0.013327719 |
| hsa_circRNA_102560 | RPS19 | 3.4828435 | 0.031135919 |
| hsa_circRNA_072025 | GUSBP1 | 3.4602056 | 0.041480092 |
| hsa_circRNA_103758 | LRBA | 3.3909703 | 0.064759981 |
| hsa_circRNA_104706 | PTK2 | 3.3403239 | 0.072038032 |
| hsa_circRNA_100913 | PICALM | 3.3325563 | 0.062158787 |
| hsa_circRNA_100017 | GNB1 | 3.3298119 | 0.116320237 |
| hsa_circRNA_013055 | GNG5 | 3.3061603 | 0.197957136 |
| hsa_circRNA_071106 | ARHGAP10 | 3.25252 | 0.061327688 |
| hsa_circRNA_103176 | GUSBP11 | 3.24701 | 0.026063829 |
| hsa_circRNA_104103 | ZFAND3 | 3.2405407 | 0.152037495 |
| hsa_circRNA_406828 | CDC40 | 3.2225694 | 0.171080612 |
| hsa_circRNA_100632 | SAMD8 | 3.2167432 | 0.180622247 |
| hsa_circRNA_104821 | FAM120A | 3.2007423 | 0.061738974 |
| hsa_circRNA_104853 | RAD23B | 3.1908217 | 0.197718314 |
| hsa_circRNA_101319 | RBM23 | 3.1764601 | 0.04167113 |
| hsa_circRNA_103881 | GUSBP9 | 3.1170457 | 0.02044704 |
| hsa_circRNA_075896 | GUSBP2 | 3.1163025 | 0.074975094 |
| hsa_circRNA_402113 | SYMPK | 3.1002052 | 0.096303139 |
| hsa_circRNA_407176 | ZCCHC7 | 3.0882944 | 0.180077993 |
| hsa_circRNA_102402 | DAZAP1 | 3.079132 | 0.194936423 |
| hsa_circRNA_104545 | DNAJB6 | 3.0788998 | 0.033931794 |
| hsa_circRNA_100583 | ARHGAP12 | 3.0622707 | 0.096050977 |
| hsa_circRNA_100019 | GNB1 | 3.0550777 | 0.222519386 |
| hsa_circRNA_046843 | ANKRD12 | 3.0451581 | 0.088286381 |
| hsa_circRNA_000166 | RPPH1 | 3.0380204 | 0.124023464 |
| hsa_circRNA_030162 | TPT1 | 3.0295003 | 0.262971671 |
| hsa_circRNA_100311 | MAN1A2 | 3.0131396 | 0.125115748 |
| hsa_circRNA_103514 | FNDC3B | 2.9802803 | 0.100027864 |
| hsa_circRNA_023928 | PICALM | 2.9579739 | 0.094229595 |
| hsa_circRNA_087856 | RAD23B | 2.953404 | 0.221451689 |
| hsa_circRNA_083171 | DNAJB6 | 2.922077 | 0.044486109 |
| hsa_circRNA_103223 | DDX17 | 2.9090677 | 0.18818366 |
| hsa_circRNA_103871 | SMA4 | 2.9053403 | 0.040002333 |
| hsa_circRNA_104707 | PTK2 | 2.9051537 | 0.038355379 |
| hsa_circRNA_104128 | GUSBP4 | 2.8874622 | 0.057598576 |
| hsa_circRNA_103559 | UBXN7 | 2.8863916 | 0.035379853 |
| hsa_circRNA_103657 | MTHFD2L | 2.8841437 | 0.138220943 |
| hsa_circRNA_100914 | PICALM | 2.8825813 | 0.209238159 |
| hsa_circRNA_104565 | PSD3 | 2.8816793 | 0.039755941 |
| hsa_circRNA_039187 | HERC2P4 | 2.8809903 | 0.035309129 |
| hsa_circRNA_104700 | PTK2 | 2.8798707 | 0.15795154 |
| hsa_circRNA_104645 | STAU2 | 2.8778354 | 0.070535377 |
| hsa_circRNA_051244 | RPS19 | 2.8698864 | 0.034479067 |
| hsa_circRNA_092437 | POLR2A | 2.8527478 | 0.183992748 |
| hsa_circRNA_104001 | RARS | 2.837513 | 0.028486195 |
| hsa_circRNA_080968 | ADAM22 | 2.8332063 | 0.098900481 |
| hsa_circRNA_051077 | AKT2 | 2.8015178 | 0.172767884 |
| hsa_circRNA_104820 | FAM120A | 2.7929752 | 0.063348674 |
| hsa_circRNA_100456 | KCNK2 | 2.7596755 | 0.069200594 |
| hsa_circRNA_104705 | PTK2 | 2.720641 | 0.057731996 |
| hsa_circRNA_074712 | LARP1 | 2.7177877 | 0.22398687 |
| hsa_circRNA_103470 | PLXND1 | 2.7175419 | 0.158807935 |
| hsa_circRNA_007352 | AKAP17A | 2.7162555 | 0.225501771 |
| hsa_circRNA_104811 | NTRK2 | 2.6995402 | 0.10624587 |
| hsa_circRNA_101836 | NFATC3 | 2.6983904 | 0.096833299 |
| hsa_circRNA_000625 | MYO9A | 2.6845656 | 0.019547598 |
| hsa_circRNA_104804 | UBQLN1 | 2.6792584 | 0.073770834 |
| hsa_circRNA_002144 | RPPH1 | 2.6690626 | 0.169683553 |
| hsa_circRNA_100705 | OAT | 2.6333758 | 0.029561512 |
| hsa_circRNA_007624 | BCAR3 | 2.5893405 | 0.116431638 |
| hsa_circRNA_002172 | RPPH1 | 2.5840657 | 0.235111699 |
| hsa_circRNA_007148 | FNDC3B | 2.5730806 | 0.130299986 |
| hsa_circRNA_103659 | MTHFD2L | 2.5721453 | 0.175086357 |
| hsa_circRNA_104854 | RAD23B | 2.5492808 | 0.319681549 |
| hsa_circRNA_103870 | SMA4 | 2.546841 | 0.050016968 |
| hsa_circRNA_103044 | RBM39 | 2.5466433 | 0.047890512 |
| hsa_circRNA_017516 | KLF6 | 2.5460504 | 0.210167733 |
| hsa_circRNA_016458 | KCNK2 | 2.5439845 | 0.145970528 |
| hsa_circRNA_103491 | RNF13 | 2.5344433 | 0.097375316 |
| hsa_circRNA_103102 | DIDO1 | 2.526908 | 0.107100533 |
| hsa_circRNA_104332 | MPP6 | 2.5227606 | 0.149044007 |
| hsa_circRNA_100917 | PICALM | 2.4979852 | 0.102662163 |
| hsa_circRNA_005054 | FMN1 | 2.4905471 | 0.131277593 |
| hsa_circRNA_104333 | MPP6 | 2.4893596 | 0.154624525 |
| hsa_circRNA_103572 | LRCH3 | 2.4688066 | 0.02332663 |
| hsa_circRNA_006725 | RHOBTB3 | 2.4678498 | 0.063375237 |
| hsa_circRNA_102742 | UGP2 | 2.4574279 | 0.090102695 |
| hsa_circRNA_069980 | MTHFD2L | 2.4563761 | 0.105002876 |
| hsa_circRNA_102762 | EXOC6B | 2.4558537 | 0.034357385 |
| hsa_circRNA_101050 | ARF3 | 2.4556044 | 0.187689863 |
| hsa_circRNA_104851 | RAD23B | 2.4488643 | 0.189141233 |
| hsa_circRNA_103993 | FAM114A2 | 2.448636 | 0.035842452 |
| hsa_circRNA_104334 | MPP6 | 2.4461134 | 0.149217116 |
| hsa_circRNA_004225 | GSK3B | 2.4365379 | 0.221844537 |
| hsa_circRNA_016771 | OBSCN | 2.4251631 | 0.178042488 |
| hsa_circRNA_079614 | MPP6 | 2.4211736 | 0.164013056 |
| hsa_circRNA_100720 | DOCK1 | 2.4201673 | 0.116328839 |
| hsa_circRNA_016459 | KCNK2 | 2.4112912 | 0.149259035 |
| hsa_circRNA_103045 | RBM39 | 2.404908 | 0.05728963 |
| hsa_circRNA_031235 | RBM23 | 2.3987064 | 0.022662274 |
| hsa_circRNA_100912 | PICALM | 2.3985638 | 0.29899446 |
| hsa_circRNA_101066 | SCN8A | 2.3956272 | 0.233804784 |
| hsa_circRNA_103469 | COPG1 | 2.3942407 | 0.114271854 |
| hsa_circRNA_089837 | AK000470 | 2.383897 | 0.109105258 |
| hsa_circRNA_101153 | ACAD10 | 2.3823245 | 0.002609931 |
| hsa_circRNA_033149 | SETD3 | 2.3780587 | 0.065861863 |
| hsa_circRNA_002111 | MED13L | 2.3724351 | 0.055507748 |
| hsa_circRNA_104000 | RARS | 2.3694198 | 0.100008903 |
| hsa_circRNA_104709 | PTK2 | 2.3644035 | 0.081573352 |
| hsa_circRNA_105023 | MID2 | 2.3587129 | 0.160922803 |
| hsa_circRNA_103046 | RBM39 | 2.3565089 | 0.096517899 |
| hsa_circRNA_100919 | PICALM | 2.3557706 | 0.193823027 |
| hsa_circRNA_001177 | NRIP1 | 2.348821 | 0.213033319 |
| hsa_circRNA_100354 | DAP3 | 2.3305058 | 0.038452486 |
| hsa_circRNA_100422 | DENND1B | 2.3297145 | 0.105045659 |
| hsa_circRNA_102294 | ANKRD12 | 2.3249592 | 0.115111777 |
| hsa_circRNA_101816 | FTO | 2.3225561 | 0.168680146 |
| hsa_circRNA_103682 | SEC31A | 2.3214319 | 0.041999543 |
| hsa_circRNA_400661 | CACUL1 | 2.3211222 | 0.257312578 |
| hsa_circRNA_100718 | DOCK1 | 2.3178226 | 0.131818921 |
| hsa_circRNA_404701 | ZNF124 | 2.3157739 | 0.125208811 |
| hsa_circRNA_103756 | LRBA | 2.3150918 | 0.044565698 |
| hsa_circRNA_103704 | PDLIM5 | 2.3113516 | 0.044615883 |
| hsa_circRNA_001312 | CCDC66 | 2.2866289 | 0.130615846 |
| hsa_circRNA_104766 | UBAP2 | 2.2822014 | 0.135075921 |
| hsa_circRNA_007691 | FAM114A2 | 2.2807603 | 0.035858164 |
| hsa_circRNA_081379 | ZKSCAN1 | 2.2686428 | 0.119044124 |
| hsa_circRNA_007927 | GTF2IP1 | 2.2629201 | 0.121400396 |
| hsa_circRNA_100016 | GNB1 | 2.2606699 | 0.186136902 |
| hsa_circRNA_077109 | IRAK1BP1 | 2.2537477 | 0.06093731 |
| hsa_circRNA_101493 | TMEM87A | 2.2470114 | 0.064184718 |
| hsa_circRNA_103830 | HMGCS1 | 2.2357729 | 0.180503269 |
| hsa_circRNA_101292 | PCCA | 2.2323362 | 0.133919052 |
| hsa_circRNA_104137 | EEF1A1 | 2.2307796 | 0.120227281 |
| hsa_circRNA_103475 | RYK | 2.2255857 | 0.08179455 |
| hsa_circRNA_103757 | LRBA | 2.2227327 | 0.049589417 |
| hsa_circRNA_100915 | PICALM | 2.2187619 | 0.251848902 |
| hsa_circRNA_104791 | PCSK5 | 2.2174512 | 0.09721482 |
| hsa_circRNA_001691 | HNRNPA2B1 | 2.2167913 | 0.288877941 |
| hsa_circRNA_103348 | SCAP | 2.2082699 | 0.170984942 |
| hsa_circRNA_005855 | TPM4 | 2.2076364 | 0.062210224 |
| hsa_circRNA_074166 | CTNNA1 | 2.206085 | 0.15718056 |
| hsa_circRNA_104469 | SND1 | 2.2031785 | 0.181746721 |
| hsa_circRNA_101436 | SETD3 | 2.2004564 | 0.039193302 |
| hsa_circRNA_104808 | UBQLN1 | 2.1974073 | 0.193070519 |
| hsa_circRNA_001218 | AP1B1 | 2.192383 | 0.248770605 |
| hsa_circRNA_103252 | ATXN10 | 2.1860456 | 0.060846909 |
| hsa_circRNA_000787 | TRIM25 | 2.1712874 | 0.202081465 |
| hsa_circRNA_103695 | KLHL8 | 2.1692789 | 0.112528122 |
| hsa_circRNA_403150 | CCNI | 2.1485725 | 0.253516795 |
| hsa_circRNA_103383 | MAPKAPK3 | 2.1450862 | 0.173045295 |
| hsa_circRNA_018315 | MAPK8 | 2.1417784 | 0.22240736 |
| hsa_circRNA_023304 | CCND1 | 2.1388671 | 0.138843357 |
| hsa_circRNA_083957 | ASH2L | 2.1368379 | 0.066875037 |
| hsa_circRNA_100015 | GNB1 | 2.1332683 | 0.284329737 |
| hsa_circRNA_104823 | FAM120A | 2.1325297 | 0.058300277 |
| hsa_circRNA_092498 | RHBDD1 | 2.1286839 | 0.110365562 |
| hsa_circRNA_003956 | RNF13 | 2.119173 | 0.078655347 |
| hsa_circRNA_000760 | MLLT6 | 2.1176218 | 0.199486421 |
| hsa_circRNA_103902 | HOMER1 | 2.1103706 | 0.10190322 |
| hsa_circRNA_103140 | PDXK | 2.1012206 | 0.128338216 |
| hsa_circRNA_103536 | AP2M1 | 2.0949858 | 0.106585261 |
| hsa_circRNA_007773 | FAM114A2 | 2.0903335 | 0.034643797 |
| hsa_circRNA_403471 | ARHGAP26 | 2.0881085 | 0.062892627 |
| hsa_circRNA_075549 | RREB1 | 2.0871183 | 0.21398061 |
| hsa_circRNA_103654 | ANKRD17 | 2.0854156 | 0.230648382 |
| hsa_circRNA_100922 | PICALM | 2.079448 | 0.22118253 |
| hsa_circRNA_006473 | ARHGAP10 | 2.0785766 | 0.245624543 |
| hsa_circRNA_103681 | SEC31A | 2.0767671 | 0.048802036 |
| hsa_circRNA_101903 | BANP | 2.0762259 | 0.148012401 |
| hsa_circRNA_092547 | SMARCA5 | 2.0759676 | 0.065850431 |
| hsa_circRNA_086160 | RPL8 | 2.0719687 | 0.107472736 |
| hsa_circRNA_100442 | LPGAT1 | 2.0714085 | 0.159450287 |
| hsa_circRNA_104761 | UBAP2 | 2.0569782 | 0.125459861 |
| hsa_circRNA_063313 | DDX17 | 2.0561963 | 0.161567983 |
| hsa_circRNA_002922 | ZNF124 | 2.0539167 | 0.045782301 |
| hsa_circRNA_050898 | ACTN4 | 2.0532008 | 0.245820548 |
| hsa_circRNA_101533 | SLTM | 2.0503113 | 0.062993395 |
| hsa_circRNA_104327 | FAM126A | 2.0500604 | 0.028734123 |
| hsa_circRNA_032969 | TC2N | 2.0495168 | 0.223090549 |
| hsa_circRNA_102072 | STAT5B | 2.048694 | 0.285431142 |
| hsa_circRNA_401901 | FASN | 2.0407444 | 0.073124623 |
| hsa_circRNA_092554 | RARS | 2.0404085 | 0.118747399 |
| hsa_circRNA_002102 | RPL14 | 2.0360372 | 0.060832351 |
| hsa_circRNA_405159 | TDRD3 | 2.0323705 | 0.018165441 |
| hsa_circRNA_031900 | TMX1 | 2.0306381 | 0.13723007 |
| hsa_circRNA_100190 | FOXJ3 | 2.0203636 | 0.055126608 |
| hsa_circRNA_403530 | GMDS | 2.0196056 | 0.213380332 |
| hsa_circRNA_100722 | DOCK1 | 2.0188575 | 0.11564839 |
| hsa_circRNA_103829 | HMGCS1 | 2.0143014 | 0.363037719 |
| hsa_circRNA_000286 | CAPRIN1 | 2.0136467 | 0.29247865 |
| hsa_circRNA_028861 | RPLP0 | 2.0133554 | 0.290517663 |
| hsa_circRNA_101809 | LONP2 | 2.0111935 | 0.089571334 |
| hsa_circRNA_061481 | EVA1C | 2.0078597 | 0.094606553 |
| hsa_circRNA_404643 | PIK3C2B | 2.0055479 | 0.118198168 |
| hsa_circRNA_092458 | EEF2 | 2.0048095 | 0.30812732 |
