# Supplementary material for: Exosomal circRNAs contribute to intestinal development via the VEGF signalling pathway in human term and preterm colostrum
Source: Aging (Albany NY). 2021 Apr 4;13(8):11218–33. doi: 10.18632/aging.202806 (PMC8109075; doi:10.18632/aging.202806)
Supplement: Supplementary Table 2 [file aging-13-202806-s002.doc]

**Supplementary Table 2.** **The most differentially expressed circRNAs (fold change＞2.0, P value＜0.05)were theoretically bound to miRNAs.**

| **FC (abs)** | **P-value** | **Regulation** | **GeneSymbol** | **circRNA** | **MRE1** | **MRE2** | **MRE3** | **MRE4** | **MRE5** |
| --- | --- | --- | --- | --- | --- | --- | --- | --- | --- |
| 9.0734664 | 0.028059514 | up | NFATC1 | hsa_circRNA_405708 | hsa-miR-4481 | hsa-miR-6875-3p | hsa-miR-4745-5p | hsa-miR-6827-3p | hsa-miR-4722-5p |
| 2.0487171 | 0.006678272 | up | CDK14 | hsa_circRNA_104423 | hsa-miR-301a-5p | hsa-miR-371a-3p | hsa-miR-29b-1-5p | hsa-miR-221-5p | hsa-miR-372-3p |
| 2.4882464 | 0.023499948 | up | ATF7IP | hsa_circRNA_101018 | hsa-miR-338-3p | hsa-miR-1271-3p | hsa-miR-489-3p | hsa-miR-582-5p | hsa-miR-508-5p |
| 2.7290675 | 0.045014518 | up | UBA2 | hsa_circRNA_102533 | hsa-miR-215-3p | hsa-miR-92a-1-5p | hsa-miR-432-5p | hsa-miR-144-5p | hsa-miR-658 |
| 3.6149661 | 0.028188721 | up | EFTUD2 | hsa_circRNA_044097 | hsa-miR-1909-3p | hsa-miR-4711-5p | hsa-miR-6722-3p | hsa-miR-6735-5p | hsa-miR-939-3p |
| 5.0877751 | 2.73252E-05 | up | GADD45A | hsa_circRNA_400011 | hsa-miR-296-3p | hsa-miR-146b-3p | hsa-miR-181d-3p | hsa-miR-504-3p | hsa-miR-328-5p |
| 3.2025458 | 0.018636864 | up | ENC1 | hsa_circRNA_073009 | hsa-miR-6734-3p | hsa-miR-6793-3p | hsa-miR-6760-5p | hsa-miR-6756-5p | hsa-miR-942-5p |
| 3.1915371 | 0.010000778 | up | LPXN | hsa_circRNA_100823 | hsa-miR-302a-3p | hsa-miR-103a-2-5p | hsa-miR-520d-3p | hsa-miR-373-3p | hsa-miR-302c-3p |
| 3.1836116 | 0.003370229 | up | CABLES1 | hsa_circRNA_102321 | hsa-miR-383-5p | hsa-miR-212-3p | hsa-miR-604 | hsa-miR-24-3p | hsa-miR-130b-5p |
| 3.0962513 | 0.000146573 | up | AP4M1 | hsa_circRNA_104438 | hsa-miR-637 | hsa-miR-647 | hsa-miR-485-5p | hsa-miR-628-5p | hsa-miR-93-3p |
| 3.0711469 | 0.00665509 | up | SOCS2-AS1 | hsa_circRNA_027719 | hsa-miR-6809-3p | hsa-miR-942-5p | hsa-miR-4469 | hsa-miR-1343-3p | hsa-miR-6787-3p |
| 3.0677124 | 0.000889646 | up | ATP1B3 | hsa_circRNA_402980 | hsa-miR-3145-3p | hsa-miR-382-3p | hsa-miR-548e-3p | hsa-miR-4652-3p | hsa-miR-20b-5p |
| 2.9559398 | 0.043684434 | up | MLLT3 | hsa_circRNA_104743 | hsa-miR-136-5p | hsa-miR-335-3p | hsa-miR-608 | hsa-miR-639 | hsa-miR-762 |
| 2.8499296 | 0.001446797 | up | RAB40C | hsa_circRNA_101672 | hsa-miR-874-3p | hsa-miR-766-3p | hsa-miR-675-5p | hsa-miR-187-3p | hsa-miR-492 |
| 2.8309804 | 0.001368584 | up | PPP1R12A | hsa_circRNA_001676 | hsa-miR-15b-3p | hsa-miR-627-3p | hsa-miR-208a-5p | hsa-miR-29b-1-5p | hsa-miR-96-3p |
| 2.8162426 | 0.010782514 | up | CD99L2 | hsa_circRNA_091692 | hsa-miR-4529-5p | hsa-miR-876-3p | hsa-miR-4480 | hsa-miR-6764-3p | hsa-miR-4685-5p |
| 2.7820591 | 0.014540657 | up | ANKRD12 | hsa_circRNA_102296 | hsa-miR-578 | hsa-miR-429 | hsa-miR-449a | hsa-miR-449b-5p | hsa-miR-34c-5p |
| 2.7627001 | 0.022996403 | up | ATP5SL | hsa_circRNA_051239 | hsa-miR-6867-5p | hsa-miR-574-5p | hsa-miR-6799-5p | hsa-miR-3162-5p | hsa-miR-4739 |
| 2.7274333 | 0.006302046 | up | ZBTB25 | hsa_circRNA_101366 | hsa-miR-30d-5p | hsa-miR-30a-5p | hsa-miR-424-5p | hsa-miR-30e-5p | hsa-miR-377-3p |
| 2.6854109 | 0.028851265 | up | TET3 | hsa_circRNA_006251 | hsa-miR-4763-3p | hsa-miR-4688 | hsa-miR-1207-5p | hsa-miR-6743-5p | hsa-miR-328-5p |
| 2.0807202 | 0.027201666 | down | C17orf53 | hsa_circRNA_004239 | hsa-miR-497-5p | hsa-miR-424-5p | hsa-miR-6838-5p | hsa-miR-450a-2-3p | hsa-miR-15b-5p |
| 2.1930395 | 0.018538341 | down | BRCA2 | hsa_circRNA_029929 | hsa-miR-6756-5p | hsa-miR-5002-5p | hsa-miR-8083 | hsa-miR-6874-5p | hsa-miR-6858-3p |
| 2.2067187 | 0.035887351 | down | KIAA1429 | hsa_circRNA_084900 | hsa-miR-4778-3p | hsa-miR-877-3p | hsa-miR-6881-3p | hsa-miR-5196-3p | hsa-miR-6809-3p |
| 2.3433843 | 0.006040221 | down | OSBPL9 | hsa_circRNA_400229 | hsa-miR-6868-3p | hsa-miR-1237-3p | hsa-miR-6792-3p | hsa-miR-7844-5p | hsa-miR-508-3p |
| 2.0140925 | 0.023535815 | down | SMARCC1 | hsa_circRNA_103356 | hsa-miR-103a-2-5p | hsa-miR-29b-1-5p | hsa-miR-136-5p | hsa-miR-17-3p | hsa-miR-1271-3p |
| 20.6594208 | 0.044982909 | down | SCRIB | hsa_circRNA_001831 | hsa-miR-3151-5p | hsa-miR-6791-5p | hsa-miR-939-5p | hsa-miR-637 | hsa-miR-7974 |
| 11.8597859 | 0.045220758 | down | HSPG2 | hsa_circRNA_010575 | hsa-miR-661 | hsa-miR-4763-3p | hsa-miR-3620-5p | hsa-miR-6791-5p | hsa-miR-939-5p |
| 5.7194571 | 0.041322837 | down | MYO9B | hsa_circRNA_102471 | hsa-miR-636 | hsa-miR-637 | hsa-miR-634 | hsa-miR-597-3p | hsa-miR-15a-5p |
| 4.3578619 | 0.00837232 | down | RHOBTB3 | hsa_circRNA_007444 | hsa-miR-4778-3p | hsa-miR-4773 | hsa-miR-6875-3p | hsa-miR-424-5p | hsa-miR-3978 |
| 4.267976 | 0.043037677 | down | RPPH1 | hsa_circRNA_006853 | hsa-miR-7974 | hsa-miR-3680-5p | hsa-miR-6750-3p | hsa-miR-627-5p | hsa-miR-6753-3p |
| 4.2004572 | 0.042253083 | down | WWC3 | hsa_circRNA_089866 | hsa-miR-1914-5p | hsa-miR-556-5p | hsa-miR-3692-5p | hsa-miR-4268 | hsa-miR-4640-5p |
| 3.8051867 | 0.046157007 | down | MAN1A2 | hsa_circRNA_013729 | hsa-miR-6881-3p | hsa-miR-6809-3p | hsa-miR-6875-3p | hsa-miR-4533 | hsa-miR-6853-3p |
| 3.4838673 | 0.013327719 | down | RERE | hsa_circRNA_100039 | hsa-miR-335-3p | hsa-miR-550a-3p | hsa-miR-571 | hsa-miR-105-5p | hsa-miR-377-3p |
| 3.4828435 | 0.031135919 | down | RPS19 | hsa_circRNA_102560 | hsa-miR-432-5p | hsa-miR-133a-3p | hsa-miR-133b | hsa-miR-103a-2-5p | hsa-miR-670-5p |
| 3.4602056 | 0.041480092 | down | GUSBP1 | hsa_circRNA_072025 | hsa-miR-485-3p | hsa-miR-371a-5p | hsa-miR-7156-5p | hsa-miR-6779-5p | hsa-miR-1273h-5p |
| 3.24701 | 0.026063829 | down | GUSBP11 | hsa_circRNA_103176 | hsa-miR-509-5p | hsa-miR-134-5p | hsa-miR-181b-3p | hsa-miR-194-3p | hsa-miR-887-5p |
| 3.1764601 | 0.04167113 | down | RBM23 | hsa_circRNA_101319 | hsa-miR-138-5p | hsa-miR-135a-3p | hsa-miR-338-3p | hsa-miR-135b-5p | hsa-miR-135a-5p |
| 3.1170457 | 0.02044704 | down | GUSBP9 | hsa_circRNA_103881 | hsa-miR-133a-3p | hsa-miR-133b | hsa-miR-371a-5p | hsa-miR-181b-3p | hsa-miR-632 |
| 3.0788998 | 0.033931794 | down | DNAJB6 | hsa_circRNA_104545 | hsa-miR-1298-3p | hsa-miR-345-3p | hsa-miR-1-3p | hsa-miR-1224-3p | hsa-miR-206 |
| 2.922077 | 0.044486109 | down | DNAJB6 | hsa_circRNA_083171 | hsa-miR-4633-5p | hsa-miR-455-5p | hsa-miR-4659b-3p | hsa-miR-4659a-3p | hsa-miR-196b-3p |
